# Health Care Workers' Perceptions on Factors Affecting Diabetes Self-Management Among Type 2 Diabetes Mellitus Patients in Fiji: A Qualitative Study

**DOI:** 10.3389/fpubh.2022.779266

**Published:** 2022-04-04

**Authors:** Lalesh Kumar, Masoud Mohammadnezhad

**Affiliations:** School of Public Health and Primary Care, Fiji National University, Suva, Fiji

**Keywords:** Health care workers, diabetes self-management, type 2 diabetes mellitus, qualitative study, Fiji

## Abstract

**Background:**

Type 2 Diabetes Mellitus (T2DM) is known as a major cause of mortality globally. Diabetes self-management refers to daily activities undertaken to control or reduce the impact of diabetes on health and well-being to avoid further illness. Health Care Workers' (HCWs) can assist patients to be aware of self-care and solve the challenges diabetes presents. The management of diabetes can improve once HCWs promote measures that facilitate self-care activities by providing necessary information and supporting patients' initiatives to make lifestyle changes. This study aimed to explore HCWs perceptions on factors affecting diabetes self-management among T2DM patients of Fiji.

**Methods:**

A qualitative study design was conducted to explore HCWs perceptions on factors affecting diabetes self-management using two Focus Group Discussions (FGDs) in Labasa, Fiji in 2021. The study settings were the Diabetic Hub Center, special outpatient department Labasa hospital and Nasea health Center Labasa. The study settings are located in an urban area. These health facilities provide special outpatient clinics to T2DM patients including clinics for other medical conditions. Nasea Health Center had 1,300 patients, the special outpatient's department Labasa hospital had 560 patients and the diabetic hub center Labasa had 295 patients at the time of the study. These patients either had T2DM or T2DM with other medical conditions. Purposive sampling was employed to recruit HCWs. The data were collected using an interview guide in semi-structured interviews and the interviews were audio recorded. The recorded data was transcribed verbatim and analyzed using thematic analysis process.

**Results:**

The participants were 9 HCWs with age range of 25-49 years. A counselor, a medical officer, physiotherapist, nurses and dieticians were among the HCWs. Three major themes and seven sub themes were identified. These included Theme 1- “availability of services” with the sub themes of clinical services for T2DM and other support services available for T2DM patients. Theme 2- “barriers and challenges to diabetes self-management” with the sub themes of health system factors, socioeconomic factors and health system factors. Theme 3- “Needs for diabetes management” with the sub themes resources and skilled personnel.

**Conclusion:**

The findings of this study demonstrate health system challenges such as lack of material resources and human resources compounded the factors affecting diabetes self-management. HCWs training as diabetes educators and developing policy on diabetes self-management are highly recommended to facilitate diabetes self-management.

## Background

Diabetes Mellitus (DM) is characterized by hyperglycemia as a result of an absolute or relative deficit in insulin production or action. Chronic hyperglycemia is associated with end organ damage including retina, kidney, nervous system, heart and blood vessels (1, 2). Type 2 DM (T2DM) accounts for ~90% of all DM cases with a variety of modifiable and non-modifiable risk factors Non-modifiable risk factors include age, genetic and sociodemographic factors while modifiable risk factors are unhealthy diet, obesity, physical inactivity, tobacco use and alcohol (3–5). Globally, ~462 million individuals are affected by T2DM, corresponding to 6.28% of the world's population. In addition, >1 million deaths were attributed to T2DM in 2017, ranking it as the ninth major cause of mortality (6–8). High incidence rates in Island nations in the pacific region are an indication of interaction amongst genetic factors and effect of rapid nutritional transition on these indigenous populations (6).

According to the Fiji Bureau of Statistics (9), Fiji's total population stands at 884,887. The Fiji Islands is an archipelago of over 300 Islands covering more than 18,000 square kilometers. The nature of geography constitutes significant challenges to the health service delivery to a diverse population over a large maritime region. According to Fiji Ministry of Health and Medical services (MoHMS) (10), diabetes is prevalent in Fiji and the numbers are growing. Currently almost one in every three Fijians is diagnosed with diabetes accounting for 30% of the population. There are three amputations every 24 h in Fiji and the given prevalence of kidney failure has worrying cost implications on families when dialysis occurs (11). The financial burden of non-communicable diseases (NCDs) on Fiji's health care is also alarming. Moreover, the health expenditure per person in Fiji is amongst the lowest in Pacific Island Countries (PICs). The underfunded health care system cannot cope with the high prevalence of diseases. Most medical centers with trained clinicians are based in urban centers resulting in a disadvantaged rural population (12).

Fiji has a health care system that follows one sided health approach where the HCWs determine the whole management process without considering the patients point of view, understanding and motivation for self-care (13). Morgan (12) states that the increased burden on the health care system is drastically preventing progress in tackling diabetes and its complications. Although the diabetes medications are funded by the country's health care system, stocks are often depleted and insufficient. In addition, inadequate availability of diagnostic tools such as HbA1c tests, microalbumin tests and high cost of glucosticks and scarcity of wound dressing materials for diabetic foot affect delivery of optimum health care. The procurement is often delayed because suppliers operate overseas and there is lack of funds for stockpiling.

T2DM has been identified as one of the most challenging chronic diseases to manage and since the management of diabetes is often facilitated by patients and families, self-management has become the mainstay of diabetes care (14). Diabetes self-management refers to day to day activities a person must undertake to control or reduce the impact of diabetes on their health and well- being to prevent further illness. These actions involve engaging in recommended behavioral activities such as medication adherence, being active, monitoring, reducing risks, problem solving and healthy diet, that are all necessary for the successful management of diabetes (15). Since the self-care techniques in DM require behavior modification, professional Health Care Workers' (HCWs) must assist patients to become aware of self-care and to solve the challenges diabetes presents. Moreover, the management of diabetes can improve when HCWs provide measures to promote self-care activities in patients (4). HCWs play a crucial role in facilitating patients' capability by providing necessary information and supporting patients' initiatives to make lifestyle changes (7). Addressing the challenges faced by HCWs may result in greater diabetes self-management and improved clinical outcomes among T2DM patients as diabetes self- management is influenced by a range of environment and personal factors that assist HCWs to formulate interventions to improve diabetes self-management behaviors (16). Therefore, the aim of the study was to explore the HCWs' perceptions on factors affecting diabetes self-management among T2DM patients of Labasa, Fiji.

## Materials and Methods

### Study Design and Setting

A qualitative study design was applied to explore HCWs perceptions on factors affecting diabetes self-management in the three health facilities in Macuata province of Labasa from 15th March to 15th April, 2021. The participants were chosen from the three health facilities but the FGDs were conducted at two study settings. The study setting was chosen as these are the only three health facilities that provide clinics to T2DM patients in Labasa Medical area. These health facilities were special outpatient department Labasa hospital, Diabetic Hub center Labasa and Nasea health Center, Labasa. The study was conducted in an urban area where the services are available for free. These three health facilities are among the four health facilities in Labasa medical area. Nasea Health Center is located in Labasa town where as Special Outpatient Department (SOPD) and Diabetic Hub Center is located at the Labasa Divisional Hospital. Nasea health center had 1,300 patients, special outpatient's department Labasa hospital had 560 patients and Diabetic Hub Center Labasa had 295 patients at the time of the study. These patients either had diabetes or diabetes with other medical conditions. The health facilities were chosen randomly.

### Study Sample

The study population comprised of HCWs from Labasa medical area The inclusion criteria for HCWs were doctors, nurses, dieticians, physiotherapists and counselors currently working with T2DM patients attending clinic at special outpatient Labasa, Diabetic Hub center and Nasea health center of Labasa medical area. The HCWs had at least 1 year experience. Those who had <1 year experiences' or were not willing to participate in the interviews were excluded. A total of three participants were excluded from the study. One participant was not willing to participate in the study while the other two had a busy work schedule.

Non-probability purposive sampling was used to recruit HCWs for the study. Two Focus Group Discussions (FGDs) were conducted among nine HCWs. The first four HCWs were working with special outpatient's department Labasa and the other five HCWs were working with Nasea health center and Diabetic Hub Center Labasa. These HCWs are part of multidisciplinary team that provide care to diabetes patients. Dieticians provide dietary advice and issue meal plan as required. The physiotherapist assists in mobility, provide offloading to patients with wounds, and prescribe aerobic exercise, muscle strengthening exercise and weight loss exercise. The patients are also referred to counselors if they need counseling for any stress that is affecting them. The dietician, physiotherapist and counselors from special outpatient department Labasa hospital and Nasea health center also see patients at diabetic hub center. The medical officer, nurse practitioners and nurses stay at their respective health facilities.

### Data Collection Tool

The interview guide was used to conduct FGDs with HCWs. The structured questionnaire was used to collect demographic data while the interview guide explored their knowledge and perception on diabetes self-management. The literature review and the research questions were used to formulate the demographic questionnaire and interview guide. The interview guide comprised of seven open ended questions to explore their experiences working with T2DM patients.

### Study Procedure

The HCWs were alerted to the study 1 month prior to the commencement of the research. This was done by providing flyers at the respective health facilities 1 month prior to the study. Information sheets were hand delivered to HCWs 2 weeks prior to commencement of the research which included the title, aim and the benefits of the research. The written consent was obtained prior to FGDs. The information sheet and the consent form for HCWs were only in English language. The first FGD was conducted with HCWs at the Nasea Health Center while the second FGD was conducted at the Diabetic Hub Center Labasa Hospital. The three health facilities are located close to each other in Labasa. FGDs conducted at diabetic hub center included HCWs from special outpatient department Labasa hospital and diabetic hub center Labasa. These health facilities share similar resources and ways in managing T2DM patients. Prior notice was given to HCWs who were available at the respective health facilities for FGDs. The interviews and FGDs were conducted by the first author in a room that was comfortable for study participants at each health facility. The FGD took 40-50 min. The participants were informed that the interview would be audio recorded on the day of the interview. The FGDs were conducted in English language and started with greetings and brief overview of the study to make participants comfortable. The written consent was taken and participants consented for FGDs to be audio recorded.

### Data Management and Analysis

The raw data were transcribed and coded by the researchers of this study. The principal supervisor for this study was Dr. Masoud Mohammadnezhad, Associate professor at Fiji National University while the second researcher was Nurse Practitioner Lalesh Kumar. The manual thematic analysis method was used for data analysis. Thematic analysis involves systematically identifying, organizing and looking into patterns of meaning (themes) across dataset which allowed researcher to visualize and make sense of shared or collective meaning and experiences Moreover, the thematic analysis comprised of six steps; becoming familiar with the data, generating initial codes, generating themes, reviewing themes, defining themes and finally the write up (17).

The data were transcribed verbatim on a word document by listening to the interviews. The data was coded by examining the transcribed data to identify common themes such as ideas, topic and pattern that come up repeatedly for example lack of resources and lack of support. The themes were then reviewed to ensure they were an accurate representation of the data. Lastly the various themes were renamed for easier understanding and write up was done.

### Study Rigor

The venue of the FGDs was checked to ensure a comfortable environment was maintained to conduct the interview sessions. The allocated time set for the interview sessions were convenient to the participants. The demographic questionnaires and the interview guide were checked by the principal researcher prior to the study.

### Ethical Consideration

Ethical approval was received from the Fiji National University (FNU) College Human Health Research Ethics Committee (CHHREC) and also from Fiji National Research Ethics Review Committee (FNRERC). Further approval was provided from the superintendent Labasa Hospital and Divisional medical officer Northern. Written consent was obtained from each participant and participants also consented for the data to be audio recorded. The research warranted voluntary participation of study subjects. Study subjects were reassured that the data collected did not include their names and all their information would remain confidential.

## Results

### Participant Characteristics

There were two FGDs conducted among HCWs working at Diabetic Hub Center, special outpatient department and Nasea Health Center in Labasa. The HCWs for FGDs included a medical officer, a counselor, nurses, physiotherapists and dieticians. One group consisted of five participants while another group consisted of four participants, making a total of nine participants. The demographic characteristics of FGD participants (*n* = 9) consisted of one male (11%) and eight females (89%). The participants were aged between 25 and 49 years. The experience ranged from 2 to 24 years (Table 1).

**Table 1:** Demographic data of FGD participants (*n* = 9).

**Variable**	**Frequency**
**HCWs**	
Nurses	3
Dietician	2
Physiotherapist	2
Medical officer	1
Counsellor	1
**Sex**	
Male	1
Female	8
**Age group**	
20-29 years	4
30-39 years	4
>40 years	1
**Work experience (years)**	
2-9 years	5
>10years	4

#### Themes and Sub Themes

The thematic analysis produced three themes; availability of services, barriers and challenges to T2DM management and proposed strategies. The themes are further divided into sub themes (Table 2). This section presents the thematic analysis of HCWs with participants' quotations.

**Table 2:** Themes and sub themes of HCWs.

**Themes**	**Sub themes**
Availability of services	Clinical services for T2DM
	Other support services available for T2DM
Barriers and challenges to T2DM	Health system factors
management	Socioeconomic factors Cultural factors
Needs for diabetes management	Resources
	Skilled personnel

#### Theme 1: Availability of Services

The sub themes consisted of clinical services for T2DM patients and other support services that are available for T2DM patients.

#### Clinical Services for T2DM Patients

The services available for T2DM comprises of multidisciplinary team which consists of doctors, clinic nurses, foot care nurses, dietician, physiotherapist, social workers and counselors. The dieticians, physiotherapists and counselors come to these health facilities to provide multidisciplinary care. They are based at Labasa hospital and Labasa health center.

Regarding the nursing services provided, one respondent stated;

“*Nurses provide health education, foot care, assist in calculating cardiovascular risk assessments, mental health assessment and help facilitate diabetes clinics”*. FGD 2 (a 49 year old female nurse)

Counsellors' as part of the multidisciplinary team provided social welfare support and psychological support to the T2DM patients'. Another respondent mentioned on the psychological support:

“*Counsellor provides psychological support and social support with the help of the social worker”*. FGD 1 (a 34 year old female counsellor).

Dieticians' gave dietary advice to all patients and provided meal plans and further follow ups to uncontrolled T2DM cases'. One respondent voiced on the dietary advice given:

“*Dieticians provide dietary advice through 1: 1 and group counseling including provision of meal plan”*. FGD 1(a 31 year old female dietician)

Physiotherapists assisted in mobility, offered muscle strengthening exercise and provided exercise prescriptions to T2DM patients'. Another respondent stated the aspects of physiotherapy:

“*Physiotherapists assist in foot care offloading, prescribe exercises, assess for mobility aid, home visits and prescribe home exercise program”*. FGD 1 (a 27 year old physiotherapist).

The medical officers' provided assessment and care inclusive of complications screening and pharmacological management. The final respondent elaborated on the pharmacological and non-pharmacological management:

“*From our side we do assessment of risk factors, pharmacological and non- pharmacological management of T2DM”*. FGD 1 (a 29 year old medical officer).

#### Other Support Services

The other support services that are available for T2DM patients included home visits and community visits with the help of community health workers', zone nurses, dieticians, physiotherapist, counselor, dental team and doctors. There is no active channel of communication that exists between diabetes peer groups, village health workers and health facilities

One respondent stated the other available support for T2DM:

“*There is peer group available but there is also poor communication. Community health workers' and zone nurses and doctors also provide services at public health level.”* FGD 2 (a 49 year old staff nurse).

#### Theme 2: Barriers and Challenges to T2DM Management

The findings demonstrated multiple barriers and challenges. The poor family support, financial constraints, lack of resources and strong cultural beliefs contributed to poor diabetes self-management practices. The sub themes comprised of socioeconomic factors, health system factors and cultural factors.

#### Socioeconomic Factors

HCWs came up with many patient's related factors comprising of lack of knowledge, lack of motivation and passion in beginning exercises, no time, busy schedule, difficulty in behavior change, use of herbal medicine, poor compliance to diet and exercise, communication breakdown, financial difficulty, poor family support and effect of recent Covid 19 pandemic. The findings show poor family support, financial constraints and lack of motivation to begin exercises.

One respondent mentioned the consequences of poor socioeconomic support:

“*Poor family support and financial constraints makes it difficult to afford basic needs, come to clinic and buy medication”* FGD 2 (a 49 year old Staff nurse)

Another respondent voiced on social issues:

“*Lack of knowledge, lack of motivation and passion in beginning exercises, no time and busy schedule of patients”*. FGD 1 (a 27 year old physiotherapist)

The following respondent identified the additional social aspects that impacted on patients' ability to manage their condition:

“*Recent covid 19 pandemic including flooding and cyclones has caused more problems, patients cannot come for scheduled clinics, cannot afford medications that are out of stock and their social welfare allowance is not sufficient. There is also communication breakdown due to language barrier and right information cannot go through”*. FGD 1(a 29 year old medical officer).

#### Health System Factors

The health system related factors were lack of material resources and human resources. The findings demonstrated scarcity of resources for the efficient health care delivery.

One respondent stated the lack of human and material resources:

“*Lack of equipment's, reduced supply of dressing materials for wound care. There is increased waiting time and patients usually get frustrated in addition to less staff to patient ratio at some Health Centers.”* FGD 1 (a 33 year old staff nurse).

Another respondent mentioned the lack of skilled personnel:

“*There is lack of skilled health personnel for specifically managing T2DM patients”*. FGD 2 (a 25 year old physiotherapist).

#### Cultural Factors

HCWs mentioned that some patients have strong belief in the use of herbal medicine. There is prevalence of herbal medicine use amongst T2DM patients that result in poor compliance to diet and exercise.

The respondent stated the influence of herbal medicine and the role of education level:

“*Health care workers are providing optimal care and support to the patients but it's the mindset of the patients rooted in their traditional herbal medicine and the ways of thinking that hinders the optimal care given. The education level and understanding each other is another barrier”* FGD 1 (a 27 year old physiotherapist).

Despite education on the need for compliance to medications and exercise, the patients are still substituting herbal medicines for antidiabetic medications. This respondent elaborated on behavior change:

“*Behavior change is very difficult. There is poor compliance to diet and exercise influenced by use of herbal medicine. FGD 1 (a 31 year old dietician)*.

#### Theme 3: Needs for Diabetes Management

The sub themes included resources and the skilled personnel. The findings show the significance of resources for the need for ongoing management of diabetes. There is lack of skilled personnel to offer specialized diabetes care.

#### Resources

Resources were inclusive of human resources, infrastructure, equipment's and consumables.

One respondent elaborated on lack of space and overcrowding:

“*We need to spend more time with patients that are not happening due to overcrowding; lack of space and shortage of staffs in some health facilities and this could be the reason why patients do not adhere to dietary advice”*. FGD 1 (a 31 year old dietician)

Another respondent mentioned regarding lack of resources:

“*There is need for more physiotherapists who can be available during diabetic clinics including foot care. More training needed for physiotherapists in offloading and foot care. Availability or resources such as felt paddings; mobility aids will facilitate better physiotherapy*. FGD 2 (a 27 year old physiotherapist).

#### Skilled Personnel

HCWs believed that there is need for more skilled personnel and training for existing HCWs. They believed that there should be more training provided to community health workers, more foot care trained nurses, introduction of trained diabetic educators and a podiatrist.

This respondent emphasized on the need for more training:

“*More foot care nurses to be trained including community health workers. There should be nurses as trained diabetes educators and there are none at present. There is need for more resources for foot care such as foot care instruments, felt padding, charts and pamphlets”*. FGD 2 (a 49 year old staff nurse)

## Discussion

The aim of the study was to explore HCWs perceptions on factors affecting diabetes self-management among T2DM patients of Labasa. The findings demonstrated the types of clinical and support services available to T2DM patients. In addition, the barriers and challenges to diabetes self-management comprised of health system factors, socioeconomic factors and cultural factors. Finally, the needs for diabetes management identified were the necessity of resources and skilled personnel.

### Availability of Services

The services available for T2DM comprised of the clinical services that were available at various health facilities, other support services included home visits, community outreaches including peer groups and community health workers.

The clinical services consisted of multidisciplinary team of doctors, nurses, dieticians, physiotherapists, counselors, dental therapists and pharmacists. Even though these services were provided to T2DM patients, the teams were not always available. This was due to shortage of staff, unavailability of space and busy clinics. The findings demonstrate that multidisciplinary approach should be strengthened to ensure best care is provided to patients. A study conducted by Siaw and Lee (18), report that multidisciplinary collaborative care appeared to positively impact on the clinical, humanistic and economical outcomes of patients with uncontrolled diabetes. In addition, the multidisciplinary care significantly improved HbA1c and systolic blood pressure over 3-12 months.

Moreover, the results of other support services demonstrated poor communication at public health level. This suggests that there is no active channel of communication between diabetes peer groups, village health workers and health facilities. If diabetes self-management education is strengthened at the community level, there will better access to diabetes care which will facilitate better diabetes self-management. Nurses and community health workers' can be trained as diabetes educators including monitoring and evaluation systems can be established to ensure diabetes self-management education is active. A study conducted by Spencer et al. (19) on effectiveness of community health worker led diabetes self-management education demonstrated achieving and maintaining improvements in the key outcomes. Another study conducted by Jiang et al. (20) found that community- based self-management group intervention for patients with diabetes has long term effects.

### Barriers and Challenges to Management of T2DM Patients

The major socioeconomic issues as perceived by HCWs were poor family support, financial difficulties, lack of motivation, lack of knowledge, busy schedule for patients. These findings suggest the need for socioeconomic support for T2DM patients to facilitate diabetes self-management. A study conducted by Ramal et al. (21) state that HCWs should educate and empower family members to act as enhancing factor for diabetes self-management. The HCWs should identify poor family support and come up with strategies such as family counseling and involving other stakeholders to strengthen social capital among patients. Since patients have poor financial status and poor financial support, the patients can be referred to social workers who can further assess and assist them financially. Family counseling can also be conducted to formulate ways in which financial support can be obtained. A study conducted by Vest et al. (22) show that barriers to diabetes self-management were directly related to patients' low income status and it is necessary for patients to activate social capital from multiple sources to achieve the most effective diabetes management.

Health system factors comprised of lack of human resources, lack of material resources and skilled personnel. As a result of limited supply of medications at the health facilities patients do not have constant supply of medications that may cause fluctuations in blood glucose levels. In addition, shortage of dressing materials and consumables may further result in compromised diabetes care. Lack of space and busy clinics may result in insufficient exchange of information between HCWs and patients. Other studies reported similar findings (23–25). Adequate supply of medications should be available through the current Fiji free medicine program where patients get medications for free at retail pharmacies. The dressing materials and other consumables can be included in the free medicine program as well. Funding to procure medications can be secured with donor agencies in addition to procurement by the MoHMS. The mobile health applications can be introduced that can be utilized to create education and awareness, a helpline number and diagnostic and treatment support.

Prevalent cultural factors perceived by HCWs included use of herbal medicines and patient's mindset and behavior. Despite adequate information provided to patients by HCWs patients are still resorting to herbal medicine and were non-compliance to medications, diet and other treatment regimens. The non-compliance to diet and medications can be as a result of poor financial status as they cannot afford healthy options. This seems to be influenced by cultural beliefs and opinions from the society and available social networks. These results were similar to studies conducted by Patel et al. (26) and Shiyanbola et al. (27). There is a need for massive health education and awareness campaigns regarding the importance of adhering to diabetes medication regimen while addressing possible complications of using herbal medicine (28–30). On the other hand, given the prevalence of herbal medicine use, a research can be conducted using the commonly used herbs in Fiji to see if it offers any benefit to patients.

### Needs for Diabetes Management

The proposed strategies by HCWs were focussed on resources such as material and human resources and availability of skilled personnel. Adequate space, equipment, human resource and material resources will enable HCWs to be efficient in managing diabetes and its complications. Moreover, Lack of resources may allow patients to resort to other means of treatment such as herbal medicine and prompt patients to default clinics. Similar results were seen in a study conducted by Romakin and Mohammadnezhad (25). The approaches to address lack of resources has always been challenging especially for a developing country. There is need to work closely with relevant stakeholders locally and internationally as well as liaise with donor agencies to secure ongoing support. Emphasis should be made to encourage everyone to have a health insurance. This will ensure patients are not affected by limited resources and the treatment is not delayed. Furthermore, there needs to be closer collaboration with private sector where MoHMS can finance a portion of treatment costs to eligible patients who can be consulted by private practitioners. This will reduce overcrowding and save resources.

The findings demonstrate lack of skilled personnel to provide best care for diabetes and its complications. There is need more foot care trained nurses to be available at community level. Moreover, offloading training can be provided for foot care team and physiotherapists as limited staff are available to provide offloading services to patients. This training can be offered through workshops, attachment to specialized areas including overseas attachments, formulating courses that can be offered through universities locally and internationally. In addition, secure funding from the MoHMS or donor agencies will ensure ongoing training for the HCWs.

### Limitations

The study was based in urban setting and may not fully identify social determinants of health that may be more prevalent in rural communities such as strong cultural beliefs and the remoteness that may affect the accessibility, availability and affordability of the health care. There were few difficulties in getting HCWs together as some were busy with their work schedule.

## Conclusion

The findings of FGDs conclude that services available for T2DM patients are inadequate in terms of infrastructure, skilled personnel, material resources and the poor consistency of multidisciplinary approach for T2DM at health facilities. Finally, the socioeconomic issues and cultural practices were also prevalent factors in FGDs. Future research can be conducted in rural settings as this may obtain in-depth understanding of cultural and environmental factors affecting diabetes self-management.

## Data Availability Statement

The original contributions presented in the study are included in the article/supplementary material, further inquiries can be directed to the corresponding author/s.

## Ethics Statement

The studies involving human participants were reviewed and approved by Fiji National University (FNU) College Human Health Research Ethics Committee (CHHREC). The patients/participants provided their written informed consent to participate in this study.

## Author Contributions

Data was collected, analysed, and interpreted by LK. Research proposal was guided by MM. The design of the study was undertaken by all authors. All authors contributed toward the manuscript preparation and approved the final manuscript for publication.

## Conflict of Interest

The authors declare that the research was conducted in the absence of any commercial or financial relationships that could be construed as a potential conflict of interest.

## Publisher's Note

All claims expressed in this article are solely those of the authors and do not necessarily represent those of their affiliated organizations, or those of the publisher, the editors and the reviewers. Any product that may be evaluated in this article, or claim that may be made by its manufacturer, is not guaranteed or endorsed by the publisher.
